# Process Intensification for Recombinant Marburg Virus Glycoprotein Production Using *Drosophila* S2 Cells

**DOI:** 10.1002/elsc.70022

**Published:** 2025-05-19

**Authors:** Sven Göbel, Ludwig Mayerlen, Isabelle Yazel Eiser, Lisa Fichtmueller, David Clements, Udo Reichl, Yvonne Genzel, AxelT. Lehrer

**Affiliations:** ^1^ Bioprocess Engineering Max Planck Institute for Dynamics of Complex Technical Systems, Sandtorstr Magdeburg Germany; ^2^ Department of Tropical Medicine Medical Microbiology & Pharmacology John A. Burns School of Medicine University of Hawaiʻi at Mānoa, Honolulu Hawaii USA; ^3^ Hawaii Biotech Inc, Hawaii Honolulu USA; ^4^ Bioprocess Engineering Otto‐von‐Guericke‐University Magdeburg Universitätsplatz 2 Magdeburg Germany

**Keywords:** cell culture, *Drosophila* S2, Marburg virus, process intensification, vaccine

## Abstract

*Marburg marburgvirus* (MARV) is a highly virulent human pathogen with limited therapeutic options. Recombinant MARV glycoprotein (GP) produced in *Drosophila* Schneider 2 (S2) cells has been extensively investigated as potential vaccine antigen with promising efficacy demonstrated in nonhuman primate models. However, the existing production process for MARV‐GP involving static batch cell cultures with limited scalability and process control show lower than desirable yields. Here, we assessed various process intensification strategies in single‐use orbital shaken bioreactors (OSBs) or rocking bioreactors (WAVE) and report maximum viable cell concentrations (VCCs) of 31.6 × 10^6^ cells/mL in batch, 69.5 × 10^6^ cells/mL in fed‐batch (FB), and up to 210.0 × 10^6^ cells/mL in perfusion mode. By changing from a glucose‐only feed to a CellBoost5 feed, MARV‐GP yields were increased by over two‐fold. Implementation of perfusion cultures achieved a peak MARV‐GP concentration of 57.4 mg/L and a 540% higher space‐time yield compared to the FB process in the 50 L WAVE system. However, maximum cell‐specific productivities were achieved at a VCC of 85 × 10^6^ cells/mL and decreased with increasing cell concentrations. Glycoanalysis revealed a uniform paucimannosidic *N*‐glycan profile, predominantly α‐1,6‐core‐fucosylated Man3F (F(6)M3) structures, across all production modes. Notably, transitioning pH control from CO_2_ to phosphoric acid shifted glycan profiles toward higher mannose forms, highlighting the influence of culture conditions on glycosylation.

**Abbreviations**
ATF: alternating tangential flow filtration
BF: BasicFeed
CB5: CellBoost5
EBOV: *Zaire ebolavirus*
F(6)M3: α‐1,6‐core‐fucosylated Man3F
FB: fed‐batch
GP: glycoprotein
M3: Man3
M5: Man5
MARV: *Marburg marburgvirus*
MARV‐GP: Marburg virus glycoprotein
MVD: Marburg virus disease
OSB: orbitally shaken bioreactor
p.i.: post‐induction
PES: polyethersulfone
S2: *Drosophila* Schneider 2
SB3‐XS: B10‐X OSB equipped with the 3 L modular adapter
SUDV: *Sudan ebolavirus*

## 1 Introduction

Infections with *Marburg marburgvirus* (MARV), a zoonotic filovirus, often result in Marburg virus disease (MVD) characterized by hemorrhagic fevers with fatality rates of up to 90% [1, 2]. Currently, there are no approved vaccines or specific therapeutics available. Although the rapid clinical development of viral vector vaccines during the 2013‐2016 West African outbreaks led to the approval of viral‐vectored vaccines against *Zaire ebolavirus* (EBOV) [3, 4], challenges involving temperature sensitivity [5] and safety concerns for immunocompromised individuals [6] have complicated their wide distribution and application in endemic regions. As all filoviruses express a single membrane‐bound glycoprotein (GP) on their surface and most neutralizing antibodies target GP, alternative recombinant subunit vaccine approaches based on filovirus GPs are particularly promising [1, 7]. In the development of filovirus vaccines, these recombinant subunit vaccines offer significant advantages over other approaches, such as viral vectors. Adjuvanted recombinant GPs from EBOV, *Sudan ebolavirus* (SUDV), and MARV demonstrated superior thermostability after lyophilization, eliciting immunogenicity and protective efficacy in various animal models even after extended exposure to elevated temperatures [8, 9, 10, 11]. Moreover, the administration of colyophilized multivalent formulations of multiple filovirus GPs [11] could drastically lower costs and logistical hurdles in endemic regions that are difficult to access.

**Summary**

- Existing MARV‐GP production methods face challenges due to low yields and limited scalability.
- Transitioning to fed-batch processes in orbital shaken bioreactors led to over a 2‐fold increase in viable cell concentrations and MARV‐GP yields.
- Perfusion cultures reached cell concentrations of up to 210 × 10^6^ cells/mL and achieved a 540% higher space‐time yield compared to fed‐batch in the 50 L WAVE system
- Implementing fed‐batch or perfusion cultures is crucial for overcoming production bottlenecks and reducing MARV‐GP manufacturing costs.

The MARV‐GP molecule forms a homotrimer, and previous work has shown varying success in stable expression in mammalian or insect cell lines [12]. Over the past three decades, the *Drosophila* Schneider 2 (S2) cell expression system has proven capable of producing conformationally relevant proteins for various viral vaccine targets [13], such as dengue fever virus, Japanese encephalitis virus, West Nile virus, Zika virus, Lassa virus, SARS‐CoV‐2, EBOV, SUDV, and MARV. Compared to other insect expression systems, S2 cells have several key advantages: (I) Stable integration of the expression vector carrying the of interest into the chromosome allows for high‐level expression of the protein without subsequent cell lysis (as for Baculovirus expression vector systems with Sf9, Sf21, and Hi‐5 cells) [13]. (II) Particularly for vaccine development, where speed to clinic is crucial, reduced time from DNA to protein by usage of polyclonal cell pools can significantly shorten upstream and downstream process development [14]. (III) Moreover, cell densities reached with S2 cells in batch mode typically exceed densities of other insect or mammalian cell lines by more than 10‐fold (over 50 × 10^6^ cells/mL). (IV) Lastly, S2 cells can be grown in any bioreactor used for mammalian or insect cell culture either in semiadherent or suspension mode. As the cells are very robust against changes in osmolality, shear, pH, temperature, and oxygen, production processes are usually highly robust and reproducible [13, 14, 15].

For the production of recombinant proteins used in other areas, various process intensification strategies can be employed to further increase product yields [16]. Operation in fed‐batch (FB) mode is relatively simple and often results in higher product concentrations and volumetric productivity (VP) compared to batch mode [16]. Here, toxic by‐products are typically not removed. As S2 cells seem to be insensitive toward the accumulation of such toxic by‐products [13, 14, 15], growth limitations and reduction of product concentrations as observed for, for example, mammalian cells, are not expected in FB mode [16]. Nevertheless, modern production processes typically aim to switch from classic FB to continuous perfusion mode, as even higher product concentrations and VPs can be reached in a reduced footprint [17, 18, 19]. Moreover, continuous harvest of the product shortens the residence time, which can prevent product degradation. For the implementation of perfusion, a cell retention device is required and various options have been successfully applied for insect cell cultures [20], with membrane‐based systems being usually favored due to better scalability.

In S2 cells, MARV‐GP yields are lower compared to EBOV or SUDV GPs. To address the limitations of low MARV‐GP concentrations, we assessed various process intensification strategies in orbital shaken bioreactors (OSBs) and WAVE bioreactors. Viable cell concentrations (VCCs) were increased either by application of FB strategies using different feeds or perfusion mode operation using an alternating tangential flow filtration module (ATF) equipped with a standard 0.22 µm polyethersulfone (PES) membrane. Finally, we evaluated the impact of the intensification on the glycosylation profile of the product.

## 2 Materials and Methods

### 2.1 Cell Culture and Antigen Expression

A stable recombinant monoclonal *Drosophila* S2 cell line expressing MARV‐GP was used. Here, the expression of MARV‐GP was performed essentially as previously published [7]. The cells were grown either in adherent or suspension mode at 27°C in nonbaffled 125 mL shake flasks (NEST Biotechnology, China) or 100 mm plates (Gibco, USA) in ExCell420 serum‐free medium (Sigma–Aldrich, USA) in incubators with ambient air without CO_2_. For maintenance, suspension cultures were split twice a week and adherent cultures once per week to 1.5 × 10^6^ or 1.0 × 10^6^ cells/mL, respectively.

### 2.2 Batch Production

For batch expression experiments, cells were seeded at 2.0 × 10^6^ cells/mL in 500 mL nonbaffled shake flasks (NEST Biotechnology) with 50 mL working volume (wv) and shaken at 130 rpm (3/4 inch orbital throw) at 27°C. Cells were induced 1 day post‐seeding by the addition of 0.2 M CuSO_4_ (ThermoFisher Scientific, USA) to the culture medium.

### 2.3 Fed‐Batch Production

For FB experiments, cells were seeded at 2.0 × 10^6^ cells/mL in 500 mL nonbaffled shake flasks or in an SB10‐X OSB equipped with the 3 L modular adapter (SB3‐X, Adolf Kühner AG, Switzerland) and standard 3 L single‐use bags, with 50 mL or 2.5 L wv, respectively. Cultivation conditions for shake flask FB experiments were identical as for batch experiments. Addition of concentrated feed with either glucose (25%, Sigma–Aldrich, USA), CellBoost5 (CB5, Cytiva, USA), HEKFS (Sartorius, Germany), or BasicFeed (BF, Sartorius, Germany) was started 1 day post‐induction (p.i.). The volumes of CB5, HEKFS, and BF varied from 3% to 10% (v/v) of the current culture volume and were based on the manufacturer's instructions. The addition of concentrated glucose was solely based on the current glucose concentration in the medium (measured daily) and carried out once glucose dropped below 20 mM.

Cultivations in the SB3‐X were carried out at 95 rpm (50 mm orbital throw) and 27°C. Head‐space aeration with 300 mL/min air/O_2_ (21%–80% O_2_) was used to control dissolved oxygen concentration at 50%. As for batch experiments, cells were induced 1 day post‐seeding by the addition of 0.2 M CuSO_4_.

Large‐scale production in FB mode (glucose) was carried out in WAVE bioreactors (GE Healthcare, USA) using 2 L bags (Sartorius, Germany) for N‐1 expansion and 50 L bags (GE Healthcare) for production. Inoculum production was initiated 11 days before N‐1 expansion was started. The cryopreserved cells were thawed, cultured initially in 100 mm plates (ThermoFisher Scientific) in ExCell420 medium, and expanded into 500 mL nonbaffled shake flasks to inoculate the N‐1 expansion in 2 L WAVE bags at 1.5–2.5 × 10^6^ cells/mL. Following N‐1 expansion, cells were seeded at 1.5–2.5 × 10^6^ cells/mL in 50 L WAVE bags with 5 L initial wv. After cells exceeded a VCC of 9.0 × 10^6^ cells/mL, the wv was expanded to 25 L with fresh ExCell420 medium. Cells were induced with 0.2 M CuSO_4_ (final concentration 200 µM) once VCC was above 4.0 × 10^6^ cells/mL. WAVE cultivations were carried out at 27°C with 0.15 L/min air/O_2_ flow (21%–40% O_2_), a rocking rate of 10–20 min^−1^, and an angle of 7–11. As previously mentioned, the addition of concentrated glucose was carried out as needed once glucose dropped below 20 mM.

### 2.4 Perfusion Production

Bioreactor perfusion cultivations were performed using the SB3‐X system. Seeding and process control strategies were identical as for FB experiments in the SB3‐X (see above). Cell retention was achieved by a hollow‐fiber membrane (0.2 µm PES, 65 cm, 0.15 m^2^, 1 mm lumen, Repligen, USA) connected to an ATF2 module (Repligen). Perfusion and induction were initiated 2 days post‐inoculation, once VCC was above 12 × 10^6^ cells/mL. Here, the perfusion rate was manually increased up to 1 reactor volume per day (RV/d) for Perfusion 1 and up to 2 RV/d for Perfusion 2 to prevent glucose limitation using ExCell420 medium supplemented with CuSO_4_. Peristaltic pumps (Watson Marlow 100) were used for the addition of feed and removal of permeate. Exchange flow rates of the diaphragm pump were set to 0.9 L/min, and an additional height differential of 40 cm was applied to the ATF2 controller. pH was controlled at 6.4 using either CO_2_ or 1 M phosphoric acid. The online Optura Spy biomass sensor (Aber instruments, Aberystwyth, UK) was used to monitor total biomass in real time.

### 2.5 Microsphere Immunoassay

To quantify MARV‐GP, a Luminex Microsphere Immunoassay (MIA) was performed, as previously described [21]. In brief, magnetic beads coupled to anti‐MARV GP mAb 9A11 were incubated shaking at 37°C with 1/50 diluted cell culture supernatant. To reduce matrix effects, dilutions of cell culture supernatants were performed in ExCell420 culture medium. After 2 h, the beads were washed twice and then incubated shaking for 1 h at 37°C with an R‐PE conjugated MARV‐GP‐specific mouse polyclonal IgG. Beads were washed twice and then diluted with Drive Fluid and read on a MAGPIX instrument (Diasorin, Italy). For quantification, a standard with twofold serial dilutions of previously purified MARV‐GP was made in ExCell420 medium as diluent. The standard curve consisted of 10 concentrations ranging from 4 µgl/mL to 7.8 ng/mL and was run in parallel to samples on each 96‐well plate. A logistic 5P regression model using the Luminex xPONENT software was used to interpolate the standard curve and determine concentrations for unknown samples.

### 2.6 Antigen Purification for Glycan Analysis

Following batch expression, supernatants were collected from each production run, sterile filtered (0.22 µm PES), and stored at 4°C until purification. For purification, each sample was brought to room temperature and sterile filtered (0.22 µm PES). Each sample was loaded onto a 5 mL HiTrap NHS‐Activated HP Sepharose affinity column (Cytiva) coupled with 50 mg of mAb 9A11 (Mapp Biopharmaceutical, USA). Following sample loading, the matrix was washed with PBS (pH 7.2) containing 0.05% Tween 20 followed by a second wash with PBS. MARV‐GP was eluted using 20 mM glycine buffer (pH 2.5) and buffer exchanged into sterile PBS by passage over a desalting column (HiTrap desalting ‐ Sephadex‐25; total column volume 20 mL, Cytiva). Purity and quality were assessed by SDS‐PAGE stained with Coomassie blue. MARV‐GP was concentrated using an Amicon Ultra 10K centrifugal filter (EMD Millipore, USA) and quantified by UV absorption at 280 nm. Purified MARV‐GP was stored in PBS at −80°C.

### 2.7 Capillary Gel Electrophoresis With Laser‐Induced Fluorescence Detection (CGE‐LIF) of Released *N*‐Glycans

Proteolytic digestion of purified MARV‐GP samples was performed with 20 µg of protein to support the release of *N*‐glycans, using a modified version of the previously described filter‐aided sample preparation protocol [22, 23]. After denaturation with urea (Sigma–Aldrich, Germany) and reducing steps with DL‐dithiothreitol (Sigma–Aldrich, Germany) and iodoacetamide (Sigma–Aldrich, Germany), proteinase K (New England Biolabs, USA) was added in a ratio of 1:20 and incubated overnight at 37°C. *N*‐glycans were released from glycopeptides using 1 µL PNGase F or PNGase A solution (New England Biolabs). The reaction was performed using manufacturer's provided reaction buffer. After incubation overnight at 37°C, samples were labeled with the fluorescent‐dye APTS (8‐aminopyrene‐1,3,6‐trisulfonic acid trisodium salt, Sigma–Aldrich, Germany), followed by a HILIC‐SPE cleanup using the protocol from Hennig et al. [24] and Ruhaak et al. [25]. Dried samples were dissolved in 20 µL water and diluted 1:300 in water for further measurements. For CGE‐LIF detection, 1 µL of diluted sample was measured with 0.8 µL GeneScan 500 LIZ size standard (Applied Biosystems, USA) and 0.8 µL 2nd NormMiX (glyXera, Germany) in Hi‐Di Formamide (Applied Biosystems). The CGE‐LIF measurement was performed on a 3130 Genetic Analyzer (Applied Biosystems). The system was equipped with a 4 capillary 50 cm array, filled with POP‐7 polymer (Applied Biosystems). Samples were injected at 15 kV for 5 s and separated at a voltage of 15 kV for 2800 s. Generated data were analyzed with the glycoanalysis software glyXtoolCE (glyXera). *N*‐Glycan peak annotations were confirmed by exoglycosidase digestions using α‐1,2,4,6 fucosidase O, ß‐1,4 galactosidase, and *N*‐acetylglucosaminidase S (New England Biolabs). Digestion was done overnight at 37°C in the supplied buffers. Enzyme activity was confirmed in parallel with APTS‐labeled bovine IgG (Sigma–Aldrich, Germany).

### 2.8 Offline Analytics

VCC and culture viability were measured using the Invitrogen Countess 2 automated cell counter (ThermoFisher Scientific) and trypan blue staining. Glucose concentrations were determined using the Onetouch Ultra 2 glucose blood monitor (Lifescan, USA) according to the manufacturer's instructions. The presence of MARV‐GP in clarified cell culture supernatants was confirmed by Coomassie‐stained SDS‐PAGE on 4%–12% polyacrylamide gradient gels (ThermoFisher Scientific).

### 2.9 Calculations

Cell‐specific growth rate (*µ*) and doubling time (*t*_D_) were determined using the following equations:

(1) $$\mu =\frac{\ln\left(\mathrm{VCC}(t_{n+1}\right)\;/\;\mathrm{VCC}\left(t_{n}\right)}{t_{n+1}-\;t_{n}}\;$$

(2) $$t_{D}=\frac{\ln\left(2\right)}{\mu }\;$$

Here, the VCC (cells/mL) and the cultivation time (*t* [h]) are used. For batch and FB productions, the cell broth in the bioreactor was harvested. Therefore, the MARV‐GP concentration in the bioreactor ($c_{\mathrm{MARV}-\mathrm{GP}}$ [mg/L]) and the wv (L) plus added feed volume (*V*_Feed_ [L]) were used to calculate the total yield of protein produced (Yield_B/FB_ [mg]).

(3) $$\mathrm{Yiel}d_{B/\mathrm{FB}}=c_{\mathrm{MARV}-\mathrm{GP}}\times \left(\mathrm{wv}+V_{\mathrm{Feed}}\right)$$

For perfusion cultivations, where MARV‐GP was continuously collected through the permeate, the total yield (Yield_perf_ [mg]) was calculated based on the MARV‐GP concentration in the permeate (*V*_Perm_ [L]) over the entire process time, including the final harvest.

(4) $$\mathrm{Yiel}d_{\mathrm{Perf}}=\sum_{i=0}^{i=t-1}\frac{c_{\mathrm{MARV}-\mathrm{GP},\;t+1}+c_{\mathrm{MARV}-\mathrm{GP},t}}{2}\times \left(V_{\mathrm{Perm},\;\;t+1}-V_{\mathrm{Perm},\;t}\right)$$

The VP takes into account the total yield per total medium used (*V*_tot_ [L]) and total process time (*t*_total_ [day]). For perfusion productions, the VP is solely based on the total amount of protein collected in the permeate.

(5) $$\mathrm{VP}\;=\frac{\mathrm{Yield}}{V_{\mathrm{tot}}\times t_{\mathrm{tot}}}$$

In comparison, the space‐time yield (STY) describes the yield over the total process time per wv.

(6) $$\mathrm{STY}\;=\frac{\mathrm{Yield}}{\mathrm{wv}\times t_{\mathrm{tot}}}$$

The cell‐specific productivity describes the maximum yield per cell (CSP [pg/cell]) at the highest protein concentration, whereas the productivity (*q*_p_ [pg/cell/day]) takes into account the process time. Here, the integral of viable cells (IVCs) is calculated based on the VCC over the process time.

(7) $$\mathrm{CSP}=\frac{c_{\mathrm{MARV}-\mathrm{GP},\;\max}}{\mathrm{VCC}}\;$$

(8) $$\mathrm{IVC}\;=\sum_{i=0}^{i=t-1}\frac{\mathrm{VC}C_{\;t+1}+\mathrm{VC}C_{t}}{2}\times \mathrm{wv}\times \left(\left(t+1\right)-t\right)$$

(9) $$q_{p}=\frac{\mathrm{Yield}}{\mathrm{IVC}}\;$$

## 3 Results

### 3.1 Batch Production to Establish a Benchmark Process

First, a batch experiment in a shake flask was conducted to establish a reference and benchmark for further process development. Following induction, S2 cultures grew exponentially, reaching a maximum VCC (VCC_max_) of 31.6 ± 1.1 × 10^6^ cells/mL (Table 1). The cells displayed average doubling times of 32.2 ± 4.3 h, high viabilities of 97% ± 2% until Day 7 p.i. despite growth arrest, and cell diameters ranging from 8 to 12.5 µm. pH values stayed relatively stable around 6.3 ± 0.1 throughout the entire production, while glucose was completely consumed around Day 6 p.i. Compared to noninduced S2 cells, obtained VCC_max_ was drastically reduced (48.0 × 10^6^ cells/mL, data not shown). The highest concentration of MARV‐GP (10.7 ± 1.9 mg/L) was observed at Day 3 p.i. before the onset of the stationary phase. Despite continuing cell growth and high culture viabilities, MARV‐GP concentration decreased with prolonged cultivation. At peak product concentration, a maximum VP of 2.3 ± 0.3 mg/L/day and a CSP of 0.74 ± 0.08 pg/cell was reached.

**TABLE 1:** Growth and process characteristics for shake flask MARV‐GP production in fed‐batch mode.

Feed	Total feed volume (mL)	VCC_max_ p.i. (10^6^ cells/mL)	*t*_D_ (h)	Day p.i. of peak conc	Max. MARV‐GP conc. (mg/L)	Yield (mg)	CSP (pg/cell)	VP (mg/L/day)
Control (batch)	0	31.6 ± 1.1	32.2 ± 4.3	3	10.7 ± 1.9	0.55 ± 0.08	0.74 ± 0.08	3.8 ± 0.5
Glucose	5–6	35.0 ± 3.4	37.8 ± 2.4	4	11.8 ± 2.1	0.62 ± 0.11	0.49 ± 0.14	3.2 ± 0.6
HEKFS	10	46.0 ± 2.5	37.2 ± 1.3	6	11.7 ± 0.4	0.72 ± 0.02	0.26 ± 0.02	3.3 ± 0.1
CB5	15	48.5 ± 3.5	31.6 ± 0.3	4	24.0 ± 3.6	1.44 ± 0.22	0.64 ± 0.19	6.6 ± 1.0
BF	21	69.5 ± 5.2	33.3 ± 0.4	6	16.1 ± 0.6	1.12 ± 0.11	0.24v0.01	2.9 ± 0.3

*Note:* Values are shown as mean ± STD of biological duplicates. Control in batch mode as reference.

Abbreviations: CSP, cell‐specific productivity; max. conc., maximum concentration; p.i., post‐induction; *t*_D_, doubling time; VCC_max_, maximum viable cell concentration; VP, volumetric productivity.

### 3.2 Fed‐Batch Production to Increase Product Concentration

**FIGURE 1:**
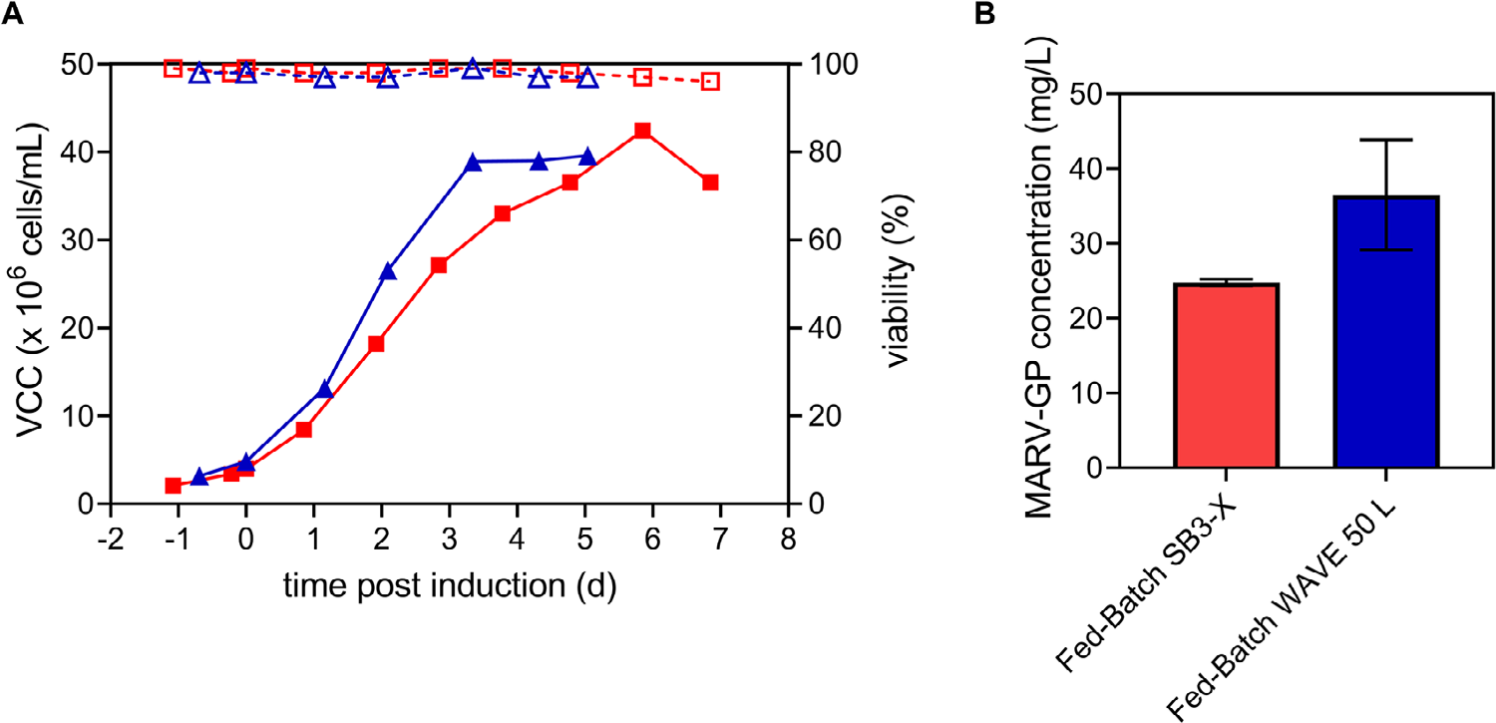
Fed‐batch single‐use bioreactor production of MARV‐GP. S2 cells were cultivated in an orbitally shaken SB3‐X bioreactor (Kuhner) (red) or 50 L WAVE bioreactor (GE Healthcare) (blue). MARV‐GP production was induced by the addition of CuSO_4_ once VCC exceeded 4.0 × 10^6^ cells/mL. 25% Glucose was fed when the glucose concentration in the medium was below 20 mM. (A) VCC (full symbols) and cell viability (empty symbols). (B) Peak MARV‐GP concentration determined by MIA. Values are represented as mean ± STD of three technical assay replicates. MARV‐GP, Marburg virus glycoprotein; MIA, Microsphere Immunoassay; S2, *Drosophila* Schneider 2; SB3‐X, SB10‐X OSB equipped with the 3 L modular adapter; VCC, viable cell concentration.

The primary reason for selecting FB mode was to mitigate nutrient limitations while achieving higher VCCs and product concentrations compared to batch mode. An established large‐scale FB production run in 50 L WAVE bags using glucose as a sole feed was directly transferred to the SB3‐X OSB bioreactor. Performance in terms of cell growth, culture viability (Figure 1A), and cell diameter was comparable between both systems. Similar cell‐specific growth rates, cell diameters ranging from 10 to 12 µm, and stable pH values of 6.4 ± 0.2 were reached for the WAVE and SB3‐X system, respectively (Figure S1). Cells cultivated in the SB3‐X system showed a prolonged growth phase p.i. (5.9 days) compared to the WAVE system (3.3 days), resulting in a higher VCC_max_ of 42.4 × 10^6^ cells/mL. Feeding with 25% glucose prevented glucose depletion, steadily maintaining glucose levels around above 18 mM (Figure S1B). Surprisingly, despite similar growth kinetics, MARV‐GP concentrations were 1.5‐fold lower in the SB3‐X system (Figure 1 B). Maximum MARV‐GP concentrations of 24.7 mg/L (Day 4 p.i.) and 36.5 mg/L (Day 5 p.i.) were reached for the SB3‐X and WAVE system, corresponding to 2.3‐ or 3.5‐fold increase compared to batch mode. As peak product concentrations were reached at lower VCCs in the SB3‐X system, CSP was slightly higher (0.75 pg/cell) compared to the WAVE system (0.69 pg/cell). Moreover, VPs were 2.4−2.8‐fold higher for both FB cultures compared to batch mode (Table 2).

**TABLE 2:** Growth and process characteristics for bioreactor MARV‐GP production in fed‐batch and perfusion mode.

	Fed‐batch SB3‐X	Fed‐batch WAVE	Perfusion 1 SB3‐X	Perfusion 2 SB3‐X
VCC_max_ p.i. (10^6^ cells/mL)	42.4	39.0	113.0	210.0
Day p.i. of peak conc.	4	5	N/A	N/A
Max. MARV‐GP conc. (mg/L)	24.7	36.5	36.9	57.4
Yield (mg)	70.7	720.5	371.0	900.2
CSP (pg/cell)	0.75	0.69	0.58	0.70
STY (mg/RV/day)	7.5	5.7	18.6	36.5
VP (mg/L/day)	6.5	5.5	2.8	2.5
Medium used (L)	2.5 + 0.1^a^	25 + 1.2^a^	16.3	36.9

Abbreviations: CSP, cell‐specific productivity; max. conc., maximum concentration; p.i., post‐induction; VCC_max_, maximum viable cell concentration; VP, volumetric productivity.

^a^ Volume of 25% glucose feed.

**FIGURE 2:**
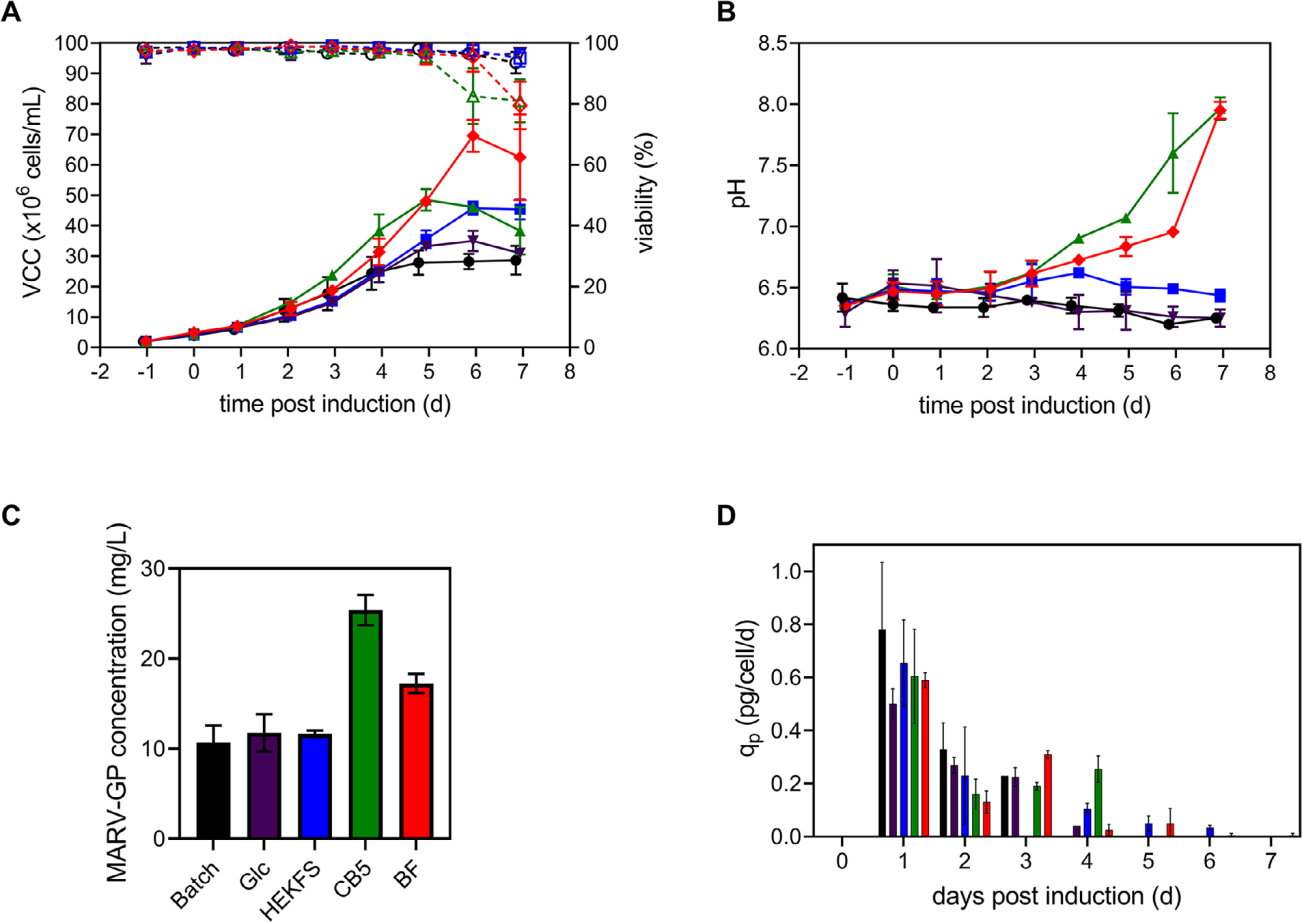
Evaluation of different feeds on MARV‐GP production in shake flasks in FB mode. HEKFS (blue), CB5 (green), and BF (red) feeds compared to glucose‐only feed (purple) and batch mode (black). S2 cells were cultivated in 500 mL non‐baffled shake flaks and protein production induced 1 day post‐seeding once VCC exceeded 4.0 × 10^6^ cells/mL. (A) VCC (full symbols) and cell viability (empty symbols). (B) pH values. (C) Peak MARV‐GP concentration determined by MIA. (D) Cell‐specific MARV‐GP production rate. Values are represented as mean ± STD of biological duplicates. CB5, CellBoost5; FB, fed‐batch; MARV‐GP, Marburg virus glycoprotein; MIA, Microsphere Immunoassay; S2, *Drosophila* Schneider 2; VCC, viable cell concentration.

Further experiments were conducted to evaluate the performance of three concentrated, chemically defined, and serum‐free feeds: CB5, HEKFS, and BF. Shake flask cultures were spiked with varying volumes of feed (Table 1) and behavior regarding cell growth, nutrient consumption, and MARV‐GP concentration were compared to feeding with glucose‐only (reference FB) or batch mode. The addition of glucose, CB5, HEKFS, or BF resulted in higher VCC_max_ compared to batch mode (Figure 2A, Table 1). Compared to feeding with glucose, a 2‐fold increase in VCC_max_ was achieved for BF and 1.3‐fold increase for CB5 and HEKFS, respectively. Over the entire growth phase similar doubling times as in batch mode were maintained for CB5 and BF (31.6 ± 0.3 and 33.3 ± 0.4 h), whereas poorer growth for feeding with glucose and HEKFS resulted in higher doubling times of 37.8 ± 2.4 and 37.2±1.3 h. Cell diameters of all cultures were comparable and ranged from 9 to 12.4 µm over the entire production (data not shown). VCC_max_ reached with glucose in shake flasks was slightly lower than scaled‐up cultivations in bioreactors (Figures 1A and 2A). The pH value of the culture medium started to increase at 3 days p.i. when feeding with CB5 and BF, whereas it remained stable for HEKFS, glucose, and batch mode (Figure 2B). For stable pH values, culture viabilities remained high throughout the entire cultivation, whereas steep declines of cell viability were observed when the pH value was approaching 7.0 (Days 5 and 6 for CB5 and BF, respectively), resulting in final culture viabilities around 80% (Figure 2A,B). Although the addition of concentrated feeds was not sufficient to keep the glucose concentration steady, glucose depletions were prevented for the HEKFS and glucose‐only feeding regimen. For CB5 and BF, cells experienced glucose depletion on Day 6 (Figure S2A). However, high cell concentrations did not necessarily translate to high MARV‐GP concentrations in the supernatant (Figure 2 C and Figure S2). Maximum MARV‐GP concentrations of 24.0 ± 3.6 mg/L were achieved for CB5, corresponding to a 2.4‐fold increase compared to batch mode, whereas the addition of BF only resulted in 1.6‐fold increases (Figure 2C). This was also reflected in the CSP, where feeding of BF resulted in the lowest productivities of 0.24 ± 0.01 pg/cell (three‐fold lower compared to batch). As for batch cultures, MARV‐GP concentrations decreased following peak concentration (Figure S2B). Interestingly, maximum cell‐specific productivities (*c*_p_) were reached 1 day p.i. for all cultures and decreased over time (Figure 2D). Scale‐down shake flask cultures fed with glucose displayed similar growth kinetics as in the SB3‐X; however, MARV‐GP concentrations were drastically lower (2.3‐fold) and in the same range as for batch cultures. Taking into account the volume increase by the addition of the respective feeds for the calculation of the total amount of MARV‐GP produced, feeding of glucose resulted in 1.1‐fold, HEKFS in 1.3‐fold, BF in 2.1‐fold, and CB5 in 2.6‐fold higher yields. A comparison of all productivity parameters for all feeds is given in Table 1.

### 3.3 Production in Perfusion Mode Allowed High Cell Concentrations and Improved Yields

Despite significant improvement in total protein yield by transition to FB mode, potential limitations by finite cell growth and protein degradation following prolonged residence times remain as only correctly folded antigen is measured by the antigen‐binding assay for MARV‐GP. Consequently, perfusion processes provide alternative means of achieving enhanced productivity without the constraints of growth restriction and high residence time. By connecting an ATF system as a cell retention device for perfusion, we were able to establish a consistent nutrient supply and, consequently, elevated VCCs, utilizing commonly applied 0.22 µm PES hollow‐fiber modules for cell retention. To characterize our system and evaluate the impact of high VCCs on MARV‐GP production and *c*_p_, two runs at different VCC set points: 100 × 10^6^ and >200 × 10^6^ cells/mL were carried out. Moreover, the applicability of a noninvasive biomass probe (Optura spy) for real‐time online monitoring of total cell concentration (TCC) was tested.

**FIGURE 3:**
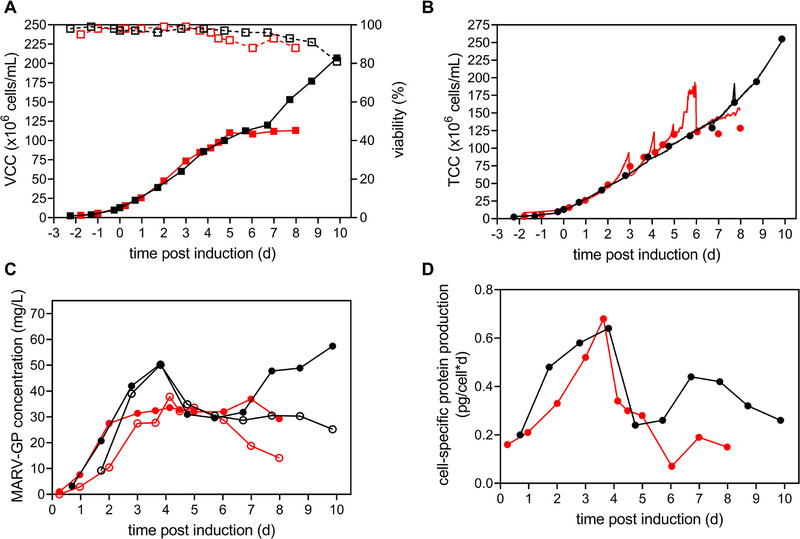
MARV‐GP production in perfusion mode using an SB3‐X connected to an ATF system. Two runs were carried out: Perfusion Run 1 (red) and perfusion Run 2 (black). Protein production was induced by the addition of CuSO_4_ once VCC exceeded 12.0 × 10^6^ cells/mL. The perfusion rate was adjusted manually over time (up to 1 RV/day for Perfusion 1 and up to 2 RV/day for Perfusion 2). For cell retention, a 0.22 µm PES hollow fiber membrane was used. (A) VCC (full symbols) and cell viability (hollow symbols). (B) Offline TCC (symbols) and online TCC (lines). (C) MARV‐GP concentration determined by MIA. Bioreactor (full symbols) and permeate (hollow symbols). (D) Cell‐specific MARV‐GP production rate. ATF, alternating tangential flow filtration; MARV‐GP, Marburg virus glycoprotein; MIA, Microsphere Immunoassay; PES, polyethersulfone; RV, reactor volume; SB3‐X, SB10‐X OSB equipped with the 3 L modular adapter; TCC, total cell concentration; VCC, viable cell concentration.

During the initial batch and subsequent perfusion phase (−2−3.5 days p.i.), the cells of perfusion Run 1 proliferated exponentially until a VCC of 73.4 × 10^6^ cells/mL with low doubling times of 24.5 h and high viabilities of 99%. Subsequently, cells continued to grow much slower (*t*_D_ of 120 h) until a VCC of 110 × 10^6^ cells/mL after which growth fully stagnated and culture viability started to decline (Figure 3A). Initially, poor growth performance after 3.5 days p.i. and subsequent stagnation was explained by fluctuations of the bioreactor wv caused by technical malfunctions of the feed pump: At Days 2, 4, and 5 p.i. malfunctions of the feed pump overnight resulted in a lack of replenishment of culture volume with fresh medium, conversely causing drastic decreases of the wv (up to two‐fold reductions) and consequently an elevated concentration of the cells within the bioreactor. After each pump malfunction, the correct wv was restored by adding fresh medium within several hours. Nevertheless, a good correlation between the reflectance signal and the TCC was obtained during the cultivation (Figure S3). By applying the correlation to the data from the online biomass probe, the equipment malfunctions could be visualized in detail by increases in TCC (Figure 3B). The most drastic example can be seen between Days 5 and 6 p.i., where the addition of fresh medium was interrupted for 12 h resulting in cell concentrations up to 190 × 10^6^ cells/mL. Despite these unintended repeated additional stress factors to the process, culture viability remained above 88% until the final harvest (Figure 3A). The MARV‐GP concentration in the bioreactor remained around 32 mg/L between Days 2 and 8 p.i. (Figure 3C). After Day 6 p.i. retention of MARV‐GP by the 0.22 PES membrane notably increased and reached values of about 50%. MARV‐GP specific production rate peaked at 0.68 pg/cell/day at 84.5 × 10^6^ cells/mL and drastically decreased with further increasing VCC (Figure 3D). Finally, a total of 0.37 g of MARV‐GP was harvested through the membrane, corresponding to an STY of 18.6 mg/RV/day and a VP of 2.8 mg/L/day (Table 2).

For perfusion Run 2, the issue of maintaining a constant wv was resolved by replacing the malfunctioning feed pump. However, the cells displayed a similar growth behavior as in the first run: An initial exponential proliferation until a VCC of 85.8 × 10^6^ cells/mL with short doubling times of 25.3 h and high viabilities of 98 % followed by retarded growth (*t*_D_ of 161 h) until a VCC of 120 × 10^6^ cells/mL. This was unexpected, as the assumed reason for the poor growth has been corrected. However, until now, pH was stably controlled at a fixed set point of 6.5 by controlled sparging of CO_2_ (Figure S4A). Starting at a VCC of 47 × 10^6^ cells/mL, an enrichment of the air stream with 10%–25% CO_2_ was required to maintain the pH set point for both runs. This resulted in a steady decline of cell growth over time (Figure S4B). For perfusion Run 1, the malfunction of the feed pump at 5 days p.i. caused a drastic spike in the sparged CO_2_ concentration, resulting in a complete growth arrest (Figure S4B). For this reason, CO_2_ was substituted with 1 M phosphoric acid at Day 7 p.i. for perfusion Run 2. Subsequently, cell growth resumed and a VCC_max_ of 210.0 × 10^6^ cells/mL was reached by Day 10 (Figure 3A). However, at the same time, the addition of phosphoric acid led to a decrease in cell viability (Figure 3A). Interestingly, this was also reflected in the MARV‐GP concentration: Compared to perfusion Run 1, a peak concentration of 50.1 mg/L was reached at Day 4 p.i., after which a steady concentration of around 30 mg/L was maintained (Figure 3C). Following the transition to phosphoric acid, MARV‐GP concentration gradually increased in the bioreactor up to 57.4 mg/L, while retention of MARV‐GP by the 0.22 PES membrane drastically increased and reached values of about 53%. Although this retention was partially responsible for the increase in the concentration in the bioreactor, *q*_p_ was also increased after the addition of phosphoric acid (Figure 3D). As for perfusion Run 1, *q*_p_ peaked at 0.64 pg/cell/day at 85.7 × 10^6^ cells/mL and drastically decreased with further increasing VCC (Figure 3D). In total 0.9 g of MARV‐GP was harvested through the membrane, corresponding to an STY of 36.5 mg/RV/day and a VP of 2.5 mg/L/day (Table 2).

### 3.4 Glycoanalysis Revealed Similar N‐Glycan Fingerprints for Various Process Modes

**FIGURE 4:**
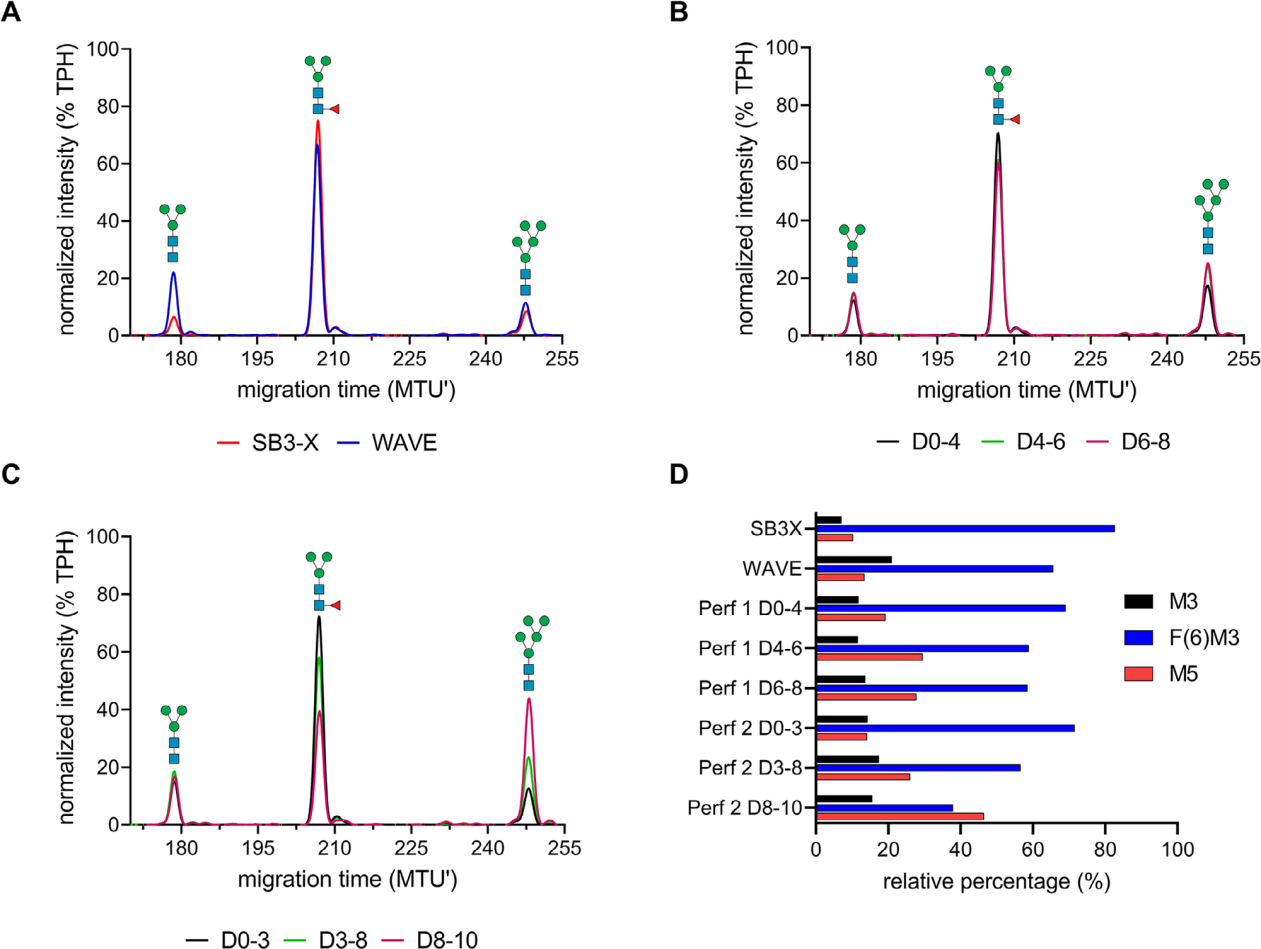
xCGE‐LIF *N*‐glycan fingerprints of MARV‐GP *N*‐glycans from different cultivations at selected time points. (A) Fed‐batch production in SB3‐X (red) or WAVE bioreactor (blue). (B) Permeate of perfusion Run 1: Days 0–4 p.i. (black), Days 4–6 p.i. (green), and Days 6–8 p.i. (pink). (C) Permeate of perfusion Run 2: Days 0–3 p.i. (black), Days 3–8 p.i. (green), and Days 8–10 p.i. (pink). (D) Relative percentage of *N*‐glycans from MARV‐GP produced in different cultivation modes. CGE‐LIF, capillary gel electrophoresis with laser‐induced fluorescence detection; MARV‐GP, Marburg virus glycoprotein; p.i., post‐induction; SB3‐X, SB10‐X OSB equipped with the 3 L modular adapter.

Analytical characterization of the *N*‐glycan profile of MARV‐GP produced in FB or perfusion mode was achieved by xCGE‐LIF (Figure 4). All samples were purified using a 5 mL HiTrap NHS‐activated HP sepharose affinity column following established laboratory scale downstream procedures used for prior vaccine antigen production. Permeate from perfusion Runs 1 and 2 was collected in multiple fractions, purified, and analyzed as individual samples. The distinct *N*‐glycan fingerprints obtained, combined with enzymatic digestion experiments (exoglycosidase digestions using α‐1,2,4,6 fucosidase O, ß‐1,4 galactosidase, and *N*‐acetylglucosaminidase S) allowed for fast and robust annotation of the *N*‐glycan structures without additional mass profiles generated with MALDI‐TOF‐MS. Relative quantification of individual *N*‐glycan structures was achieved by normalization of *N*‐glycan fingerprints to total peak height. Regardless of the production mode and stage of production (for perfusion runs), all *N*‐glycan fingerprints of MARV‐GP showed three distinct peaks at 178.4, 207.0, and 248.5 MTU. The glycans released by PNGase F or PNGase A from the S2‐cell‐produced MARV‐GP were Man3 (M3), α‐1,6‐core‐fucosylated Man3F (F(6)M3), and Man5 (M5) structures (Figure 4A–C, Figure S5), respectively. Relative quantification of these peaks revealed an abundance of F(6)M3 (6082 %), followed by M3 (7%–20%), and M5 (10%–30%). Interestingly, after switching the pH control to phosphoric acid (Days 8–10 p.i.) for perfusion Run 2, a transition from F(6)M3 toward M5 structures (up to 47%) was detected (Figure 4D).

## 4 Discussion

### 4.1 Production of MARV‐GP in Batch Mode

Observed growth characteristics of the recombinant S2 cells during antigen expression in batch mode were in line with previous reports: maximum cell densities of 16.0–50.0 × 10^6^ cells/mL, doubling times of 23–58 h, and cell sizes of 9–12 µm [15, 26, 27, 28]. As peak rates of MARV‐GP production were reached during the mid‐exponential phase, prolonged cultivation provided no benefit and instead led to product degradation as indicated by a decline in the concentration observed via conformational immunoassay. This is in stark contrast to previous studies reporting peak product concentration at the end of growth [28, 29, 30, 31], indicating a strong link of efficient MARV‐GP synthesis to exponential cell growth. Exhaustion of secondary metabolites accessory to protein folding and stabilization such as glutamine, serine, and cysteine were in other cases identified as the main causes of degradation [29]. Yet, concentrations of those were not evaluated in this study. Additionally, the observed formation of MARV‐GP aggregates could have reduced the soluble yields of MARV‐GP, interfering with the antigen‐binding assay measurement of correctly folded MARV‐GP. Due to the challenges in achieving a stable trimeric form of MARV‐GP, there have been varying degrees of success in expressing it in mammalian and insect cell lines [12]. Clarke et al. demonstrated successful expression of MARV‐GP using baculovirus‐infected Sf9 cells; however, concentrations in the supernatant were quite low (∼2 mg/L) [33]. On the other hand, the expression of MARV‐GP in HEK293T cells was not successful. Batch concentrations observed here of 10.7 ± 1.9 mg/L are in line with other antigens produced in S2 cells, such as the E‐proteins of dengue fever virus (10–50 mg/L) [34], the NS1 protein of Japanese encephalitis virus (2‐5 mg/L) [35], the E and NS1 proteins of West Nile virus (10‐25 mg/L) [36], the VAR2CSA protein (∼17 mg/L) [13], and the rabies virus GP (0.5 mg/L) [37]. Moreover, achieved *q*_p_s were higher compared to other insect cell lines such as High Five, Bm5, IPLB‐LdFB, or SF‐21 (0.05–0.21 pg/cell/day) [38].

### 4.2 First Step Toward Process Intensification: Fed‐Batch Mode Cultivation

FB mode is commonly used for the commercial production of biologics in animal cells as higher VCCs and product titers can be reached with lower volumes of media. Although WAVE bioreactor systems are an established technology utilized in preclinical, clinical, and production‐scale biotechnological facilities [39], problems related to scalability (up to 1000 L) and limited oxygen transfer via headspace aeration remain. On the other hand, the use of geometrically similar vessels facilitates the scale‐up of orbitally shaken bioreactors (from ul scale up to 2500 L). For this reason, the existing FB production process for the MARV‐GP expression cell line utilizing glucose as a sole feed was transferred to an SB3‐X bioreactor. As both systems are aerated via the headspace without a stirrer and exhibit low shear on the cells, similar growth profiles and product concentrations were expected. Compared to the single replicate SB3‐X production run, the WAVE production run (#157) was an established and thoroughly optimized GMP‐compliant process. With further optimization of the production conditions and more replicates, we are confident to match the MARV‐GP concentrations of the WAVE system in the SB3‐X system. Regardless, for both bioreactor systems, VCCs and product concentrations p.i. were increased compared to batch mode, as observed for other antigens produced in FB mode in S2 cells using glucose feed [13, 28].

Alternative feeds were identified that increased MARV‐GP production by 1.1−2.6 fold. MARV‐GP specific production rate was quite similar for batch and glucose‐fed cultures p.i., suggesting the depletion of another production‐limiting substrate (Figure 2D). While the exact composition of the various feeds is unknown, it seems that BF and CB5 contain additional substrates that positively impact MARV‐GP production, further reflected by increased q_p_’s after feeding at Days 3 and 4 p.i. Moreover, protein degradation was reduced when feeding with HEKFS and BF, reinforcing the hypothesis of depleted amino acids to be responsible for degradation. However, this was not confirmed for CB5. Additional investigations including spent medium analysis could identify critical and limiting nutrients for efficient MARV‐GP production and stability. Although S2 cells can be operated at a wide pH range (6.0–6.8) [15], it seems that an increase of pH to ∼7 in an uncontrolled system can serve as an early marker for the decline of viability and optimal harvest time point. For both, CB5 and BF, peak product concentration was reached when pH reached 7.0, followed by drastic declines in culture viability (and protein concentration for CB5) with further increase in pH.

### 4.3 Process Intensification Using an ATF Perfusion Process

Interest in integrated continuous processing of recombinant proteins has grown in recent years, as this approach enhances product quality by minimizing processing and hold times while enabling long‐term production cycles with improved VP [40]. Membrane‐based cell retention devices, such as the ATF, are commonly utilized for commercial production due to their robust performance at high cell densities and reduced product sieving compared to other membrane‐based systems [41]. Initial pH control with CO_2_ was not ideal, as CO_2_ is known to inhibit the cell growth of insect cell cultures without impacting the culture viability [42]. This was confirmed for both perfusion runs as growth rates drastically declined with increased CO_2_ concentration and increased immediately after the transition to acid‐based pH control, while culture viability remained high.

Real‐time online monitoring of VCCs is an established process analytical tool (PAT) that allows immediate adjustment of cell conditions and live insights into cell behavior and process dynamics [43]. In this context, capacitance and the online measurement of optical density are commonly used for insect cell cultures [28, 43]. Compared to traditional invasive optical density probes, which utilize transmittance or a combination of transmittance and reflectance (Dencytee, Hamilton), the OpturaSpy probe is noninvasive and exclusively measures back‐scatter/reflectance at a wavelength of 1330 nm. At this wavelength, liquid water absorbance is strong in the near‐infrared spectrum range, resulting in a well‐defined sampling area within the media since water is always present in the culture. This range maximizes sensitivity to cell biomass while minimizing interference from reflections off other particles as the wavelength penetrates the media to a maximum depth of 2.5 cm. We were able to demonstrate high linearity over a TCC range of 2–260 × 10^6^ cells/mL, comparable to the performance of optical density probes [44]. As long as the culture viability remains high (>99%), the output of the sensor can be used interchangeably for the measurement of VCC. Although online monitoring was at this time not able to prevent disturbances for perfusion Run 1, it allowed a precise temporal breakdown of the events and a retrospective analysis of the TCC. For subsequent studies, the OpturaSpy probe could, for example, be used to automate the control of a bleed rate to maintain a constant TCC throughout the production.

Starting at 2 days p.i. MARV‐GP concentration in the permeate line exceeded the maximum concentrations achieved in SB3‐X FB productions. However, productivity clearly showed a cell density effect [45] with a *q*_p_ and CSP sweet spot around 70–85 × 10^6^ cells/mL, indicating no benefit of further increasing VCC for this cell line. Despite several severe disturbances and stress factors (nutrient limitation, possible increased shear, and multiple concentrations steps), MARV‐GP production remained stable for perfusion Run 1, highlighting the robustness of the production and potential applicability for S2 cell production of vaccine antigens in lower‐resourced countries with harsh environments that typically face increased risks of disturbances, for example, power and equipment failure. At later stages of production, when culture viability decreased, MARV‐GP retention by the PES membrane increased. Nevertheless, it was possible to harvest a total amount of target protein of 371 mg for perfusion Run 1 and 900.2 mg for perfusion Run 2. Maintaining linear production kinetics by implementing a cell bleed and operating the perfusion at a steady‐state at peak *q*_p_ would allow the production of 56 mg/L/day MARV‐GP, matching the total output of a 50 L WAVE system (25 L wv) within 4.3 days while decreasing the vessel size by 10‐fold.

### 4.4 Glycoanalysis

GPs, including MARV‐GP, are highly glycosylated, and the glycosylation profile is variable depending on the expression system [12]. Regardless of the mode of production, production vessel, and stress level, the glycosylation of MARV‐GP produced in S2 cells was uniform after PNGase F or PNGase A digestion (Figure S5) and consisted mostly of paucimannosidic structures, particularly F(6)M3 glycans (60%–82%). This aligns with previously reported profiles for GPs produced in S2 cells [46, 47, 48]. Short paucimannose structures have been shown to enhance immunogenicity and could be advantageous in a vaccine where an immune response is desired [50]. Surprisingly, *N*‐glycan structures shifted toward high‐mannose structures (M5), when adding phosphoric acid to the culture for pH control. One potential explanation could be a potential direct inhibition of enzymes (e.g., α‐mannosidase II) responsible for cleavage of M5 to M3 [51], or indirect inhibition by reduced formation of activated sugars such as uridine diphosphate *N*‐acetylglucosamine both caused by the intracellular increase in phosphate. Alternatively, the addition of phosphoric acid could have decreased the processing time of the GP in the Golgi, increasing the relative amount of nonprocessed M5. The observed shift to higher mannose structures using specific process controls could offer easier opportunities for tailoring glycosylation profiles depending on the desired antigen properties compared to targeted cell line engineering or cell‐free glycosylation reactions [47, 51, 52, 53]. Vaccination of cynomolgus macaques with MARV‐GP produced in WAVE bioreactors provided complete protection against severe and lethal MVD after MARV infection [11] with potent immunogenicity of the antigen in an adjuvanted formulation. Additional in vivo studies would be required to investigate the effect of high‐mannose or complex‐type mammalian glycan structures on the immunogenicity of MARV‐GP.

## 5 Conclusion

Both process intensification strategies (FB and perfusion) outperformed the classical batch production in terms of yield. A further switch from glucose‐only to commercially available CB5 as a feed for FB processes could further increase the yield. Particularly the perfusion production is not yet fully optimized and a further assessment of production cell density and integration of a cell‐bleed might increase cell‐specific and VP. It seems that by changing between CO_2_ and phosphoric acid for pH control, cell growth and *N*‐glycan profile of the final product can be easily influenced, without the need of additional glycoengineering efforts. Finally, long‐term production (over multiple weeks) should be investigated to rule out issues with cell line stability and membrane fouling. In summary, this demonstrates that the intensified ATF‐based scalable OSB perfusion cultivation of S2 cells provides new opportunities for an economical production platform for GP‐based vaccines.

## Nomenclature

 

cp: productivity, pg/cell/day
CSP: cell‐specific productivities, pg/cell
RV: reactor volume, L
STY: space‐time yield, mg/RV/day
TCC: total cell concentration, cells/mL
*t*_D_
: doubling time, h
VCC: viable cell concentration, cells/mL
VP: volumetric productivity, mg/L/day
wv: working volume, L

### Greek Symbols

 

µ
: cell‐specific growth rate, h^−1^

## Author Contributions

S.G., Y.G., and A.T.L. developed the concept and designed the experimental strategy. S.G., L.M., I.E., L.F., and A.T.L. designed and performed the experiments, analyzed the data, and interpreted the results. S.G. wrote the original draft. All authors reviewed the data and edited the original draft.

## Ethics Statement

The authors have nothing to report.

## Conflicts of Interest

Axel T. Lehrer is named inventor on a patent covering multivalent filovirus subunit vaccines (U.S. Patent 11,433,129B2). David Clements is a current employee of Hawaii Biotech, Inc. The other authors declare no conflicts of interest.

## Supporting information



Supplementary Materials

## Data Availability

Additional data is available in the article's Supporting Information. Further data that support the findings of this study are available from the corresponding author upon reasonable request.
