# Supplementary material for: Process Intensification for Recombinant Marburg Virus Glycoprotein Production Using Drosophila S2 Cells
Source: Eng Life Sci. 2025 May 19;25(5):e70022. doi: 10.1002/elsc.70022 (PMC12087431; doi:10.1002/elsc.70022)
Supplement: Supplementary file 1 — Supplementary Materials [file ELSC-25-e70022-s001.docx]

**Journal: “Engineering in Life Sciences”**

**Manuscript Title: “Process optimization for recombinant Marburg virus glycoprotein production using Drosophila S2 cells”**

**Supplementary Material**

Figure S1 Fed-batch production of MARV-GP in a single-use bioreactor. S2 cells were cultivated in an orbitally shaken SB3-X bioreactor (Kuhner) (red) or a 50 L WAVE bioreactor (GE Healthcare) (blue). 25 % glucose was fed when the glucose concentration in the medium was below 20 mM. (A) VCC (full symbols) and cell viability (empty symbols). (B) Glucose concentration. (C) Cell-specific growth rates. (D) Cell diameter.

Figure S2 Fed-batch production of MARV-GP utilizing different feeds. HEKFS (blue square), CB5 (green triangle), and BF (red diamond) were evaluated against a glucose-only feed (purple triangle) and batch cultivation as a control (black circle). S2 cells were cultivated in 500 mL non-baffled shake flasks and protein production induced one day post seeding once VCC exceeded 4.0×10^6^ cells/mL. (A) Glucose concentration in the supernatant. (B) MARV-GP concentration over time. Values represented as mean ± STD of two biological replicates.

Figure S3 Correlation of total cell concentration (TCC) with the reflectance signal during MARV-GP production for perfusion 1 (red) and perfusion 2 (black). High correlations (R²≥0.99) were achieved over a wide TCC range (0-250x10^6^ cells/mL).

Figure S4 MARV-GP production in S2 cells in perfusion mode using an SB3-X connected to an ATF. Two runs were carried out: Perfusion 1 (red) and perfusion 2 (black). Protein production of both runs were induced by the addition of CuSO_4_ once VCC exceeded 12.0×10^6^ cells/mL. CO_2_ (perfusion 1) or CO_2_ and phosphoric acid (perfusion 2) were used for pH control. (A) pH value. The black arrow indicates the switch from CO_2_ addition to phosphoric acid for pH control. (B) Cell-specific growth rate (squares) and percentage of CO_2_ in the air supply (lines).

Figure S5 xCGE-LIF *N*-glycan fingerprints of MARV-GP *N*-glycans released after PNGase F (red) or PNGase A (blue) digestion. (A) Fed-batch production in SB3-X bioreactor. (B) Fed-batch production in WAVE bioreactor. (C) Permeate of perfusion run 2: day 3-8 p.i. (D) Permeate of perfusion run 2: day 8-10 p.i.
